# Cervical myelopathy due to subaxial calcium pyrophosphate dihydrate (CPPD) deposition with simultaneous asymptomatic crowned dens syndrome: two case reports

**DOI:** 10.1186/s12891-020-03736-x

**Published:** 2020-10-31

**Authors:** Dong-Gune Chang, Jong-Beom Park, Ho-Young Jung, Kyung Jin Seo

**Affiliations:** 1 Department of Orthopaedic Surgery, Inje University Sanggye Paik Hospital, College of Medicine, Inje University, Seoul, South Korea; 2 Department of Orthopaedic Surgery, College of Medicine, The Catholic University of Korea, Seoul, South Korea; 3 Department of Orthopaedic Surgery, Uijeongbu St. Mary’s Hospital, College of Medicine, The Catholic University of Korea, 271 Cheonbo-ro, Uijeongbu-si, Gyeonggi-do, 11765 South Korea; 4 Department of Pathology, College of Medicine, The Catholic University of Korea, Seoul, South Korea

**Keywords:** Cervical myelopathy, Subaxial calcium pyrophosphate dehydrate, Crowned dens syndrome

## Abstract

**Background:**

There are few reports of cervical myelopathy caused by an attack of subaxial calcium pyrophosphate dihydrate (CPPD) deposition. Moreover, there has been no report on cervical myelopathy by subaxial CPPD deposition with simultaneous asymptomatic crowned dens syndrome (CDS) at the same time.

**Case presentation:**

The first case was a 68-year-old male complaining of cervical myelopathic symptoms. Plain radiographs, computed tomography (CT) and magnetic resonance imaging (MRI) findings revealed spinal cord compression by calcified round lesions at C4 as well as a calcified lesion behind the dens. The second case was a 77-year-old female complaining of cervical myelopathic symptoms. Plain radiographs, CT and MRI findings revealed spinal cord compression by calcified round lesions at C3 and C4 as well as a calcified lesion behind the dens. In both cases, we believed that the calcified lesion behind the dens was an asymptomatic lesion. Therefore, the first patient received decompressive laminectomy of C3 and C4, removal of calcified round lesions, and posterior fixation from C3 to C5 due to associated kyphosis. The second patient underwent decompressive laminectomy of C3 and C4 and removal of calcified round lesions. Microscopic examination under polarized light showed dark blue calcifications with rhomboid crystals that were positively birefringent. The findings were consistent with those of CPPD.

**Conclusions:**

This is the first study to report cervical myelopathy caused by subaxial CPPD deposition with simultaneous asymptomatic CDS. Surgical removal of the subaxial CPPD deposition alone achieved a satisfactory surgical outcome without recurrence.

## Background

Calcium pyrophosphate dehydrate (CPPD) deposition is characterized by the accumulation of CPPD crystals in articular and periarticular tissues [1–7]. CPPD deposition in the cervical spine has been previously described and there are few reports of cervical myelopathy caused by subaxial CPPD deposition [8–15]. Crowned dens syndrome (CDS), which is known as retro-odontoid CPPD deposition, may usually present neck pain and stiffness and cause progressive spinal cord compression and myelopathy with disease progression [8–15].

However, to the best of our knowledge, there has been no report on cervical myelopathy caused by subaxial CPPD deposition with simultaneous asymptomatic CDS at the same time. Therefore, we hereby report two cases of cervical myelopathy caused by subaxial CPPD deposition with simultaneous asymptomatic CDS that were successfully treated by surgical removal of the subaxial CPPD deposition alone.

## Case presentation

### Case 1

**Fig. 1:**
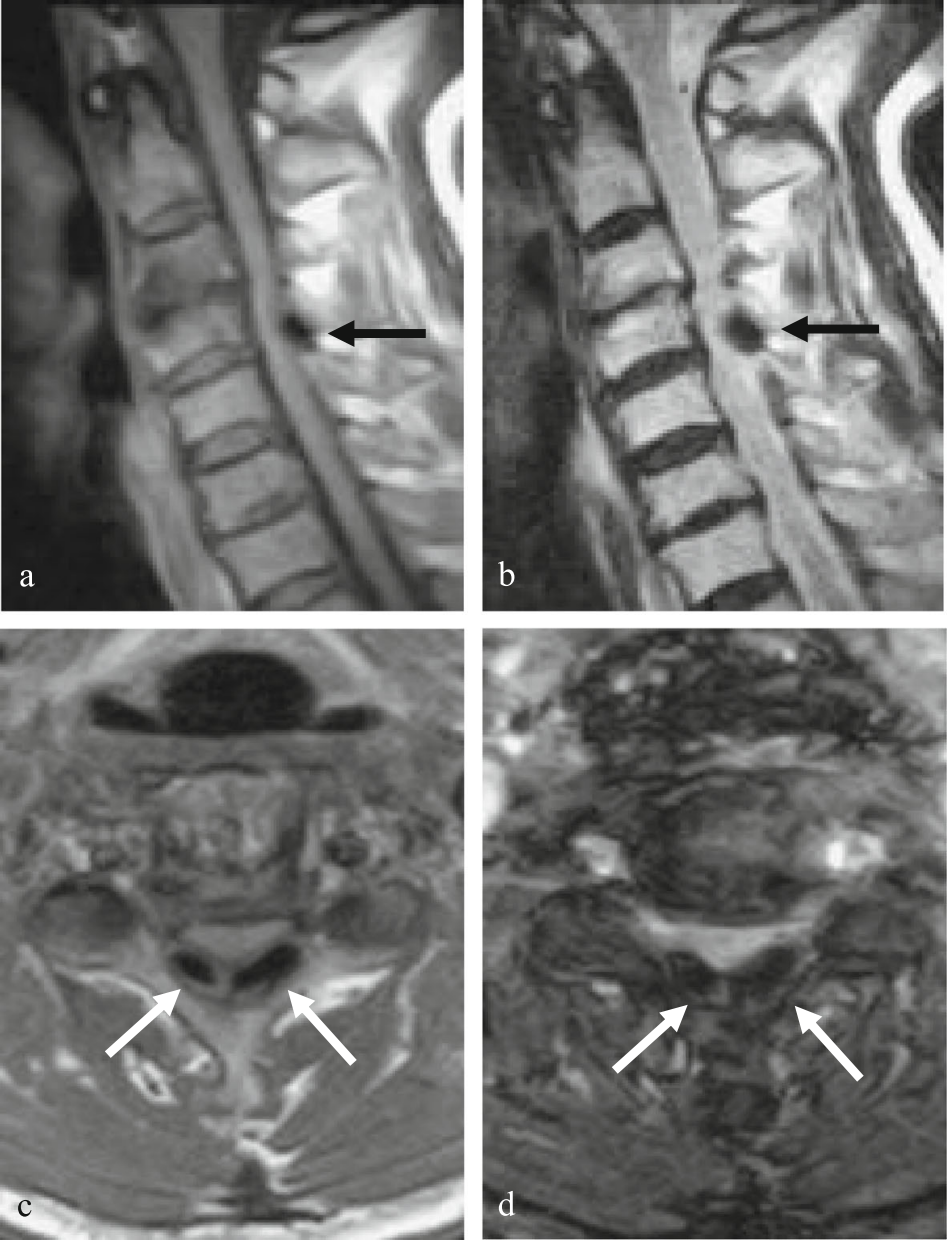
Sagittal (**a** and **b**) and axial (c and d) MRI demonstrated spinal cord compression by dark round lesions (black and white arrows) at C4 which appeared as a low signal mass on both T1 (**a** and **c**) and T2 (**b** and **d**) weighted images

**Fig. 2:**
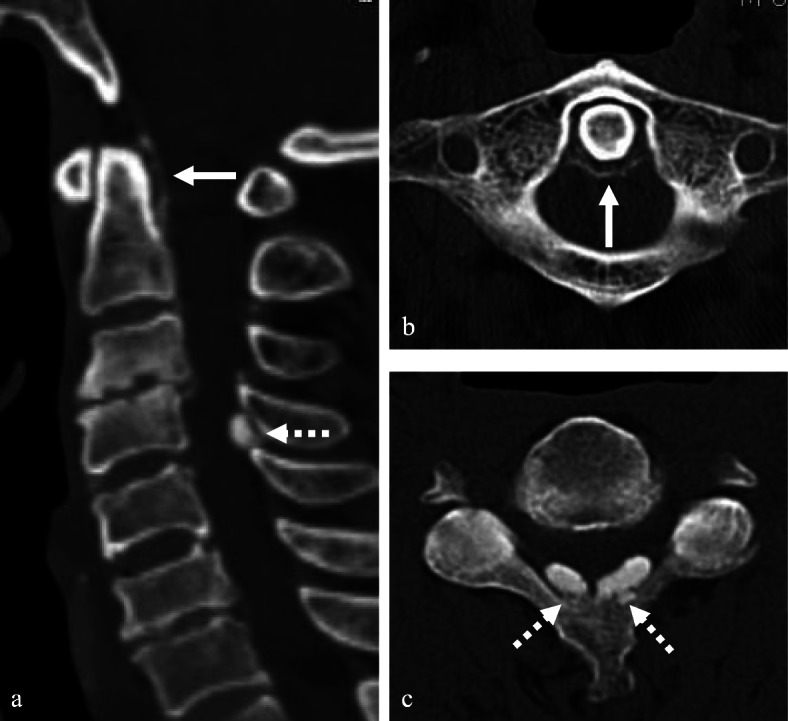
Sagittal and axial CT scans revealed calcified lesion behind the dens (**a** and **b**) (white arrows) and spinal cord compression by calcified round lesions (**a** and **c**) at C4 (dotted white arrows)

**Fig. 3:**
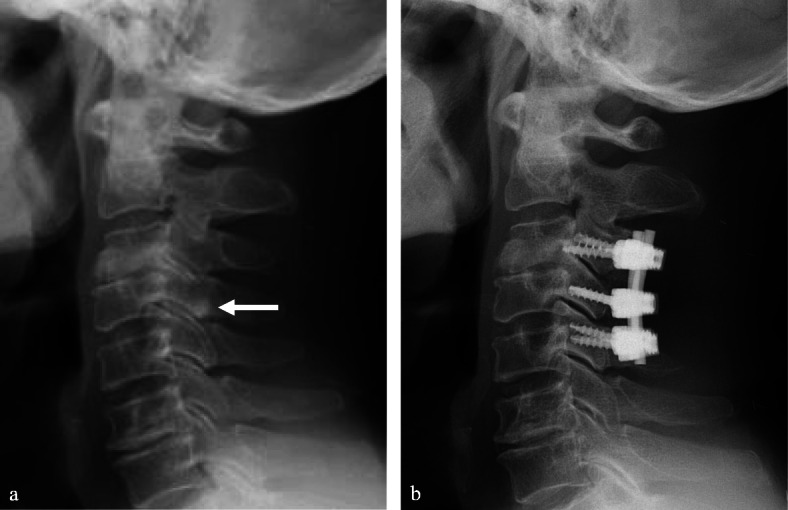
Lateral radiograph (**a**) of the cervical spine showed round calcified lesions at the lamina of C4 (white arrow) and spondylotic changes and disc space narrowing of C3-C4. Lateral radiograph (**b**) of the cervical spine showed decompressive total laminectomy of C3 and C4 and posterior fixation with lateral mass screws from C3 to C5

**Fig. 4:**
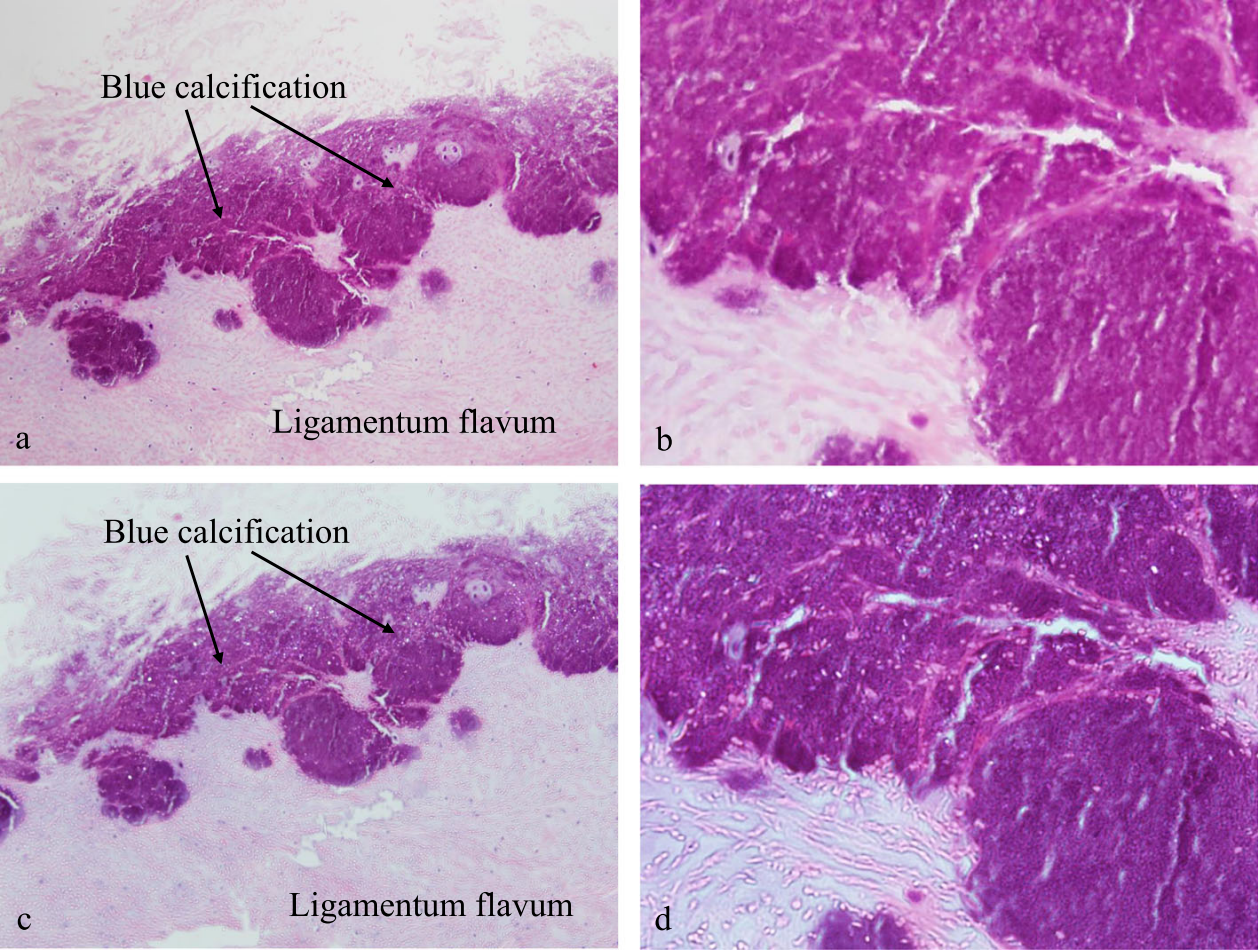
Microscopic evaluation (× 100, **a**; × 400, **b**) of the surgical specimen demonstrated degenerated ligamentum flavum with dark blue calcifications representing chondrocalcinosis. Microscopic evaluation (× 100, **c**; × 400, **d**) of the surgical specimen under polarized light showed rhomboid crystals that were positively birefringent in blue calcification area

A 68-year-old male presented with neck pain (neck visual analogue scale [VAS] score: 3), bilateral radiating arm pain (arm VAS score: 7/7), and gait disturbance for 6 weeks. He had no history of recent head or neck injuries. Neurological examination revealed a spastic gait, hand clumsiness, and exaggerated deep tendon reflexes in the bilateral upper and lower extremities. Pathologic Babinski sign and ankle clonus were present. Muscle strength of both lower extremities was decreased. Grip and release test was 18 times per 20 s. Modified Japanese Orthopaedic Association (mJOA) score was 9. However, he had no dysuria or constipation. Sagittal (Fig. 1a and b) and axial (Fig. 1c and d) magnetic resonance imaging (MRI) showed spinal cord compression by dark round lesions at C4 which appeared as a hypointense mass on both T1 and T2 weighted images. Sagittal and axial computed tomography (CT) scans showed a calcified lesion behind the dens (Fig. 2a and b) and spinal cord compression by round calcified lesions at C4 (Fig. 2a and c). Lateral radiograph of the cervical spine showed round calcified lesions at the lamina of C4 (white arrow) and spondylotic changes and disc space narrowing of C3-C4 (Fig. 3a). The calcified lesion behind the dens was thought to be an asymptomatic lesion, and the patient received decompressive laminectomy at C3 and C4, removal of round calcified lesions, and posterior fixation with lateral mass screws from C3 to C5 (Fig. 3b). Microscopic evaluation (× 100, Fig. 4a; × 400, Fig. 4b) of the surgical specimen demonstrated degenerated ligamentum flavum with dark blue calcifications representing chondrocalcinosis. Microscopic evaluation (× 100, Fig. 4c; × 400, Fig. 4d) of the surgical specimen under polarized light showed rhomboid crystals that were positively birefringent in the blue calcification area. The findings were consistent with CPPD. The patient’s myelopathic symptoms were significantly improved after surgery to mJOA of 14 with a recovery rate of 56% and showed a satisfactory surgical outcome without recurrence at a 5-year follow-up.

### Case 2

**Fig. 5:**
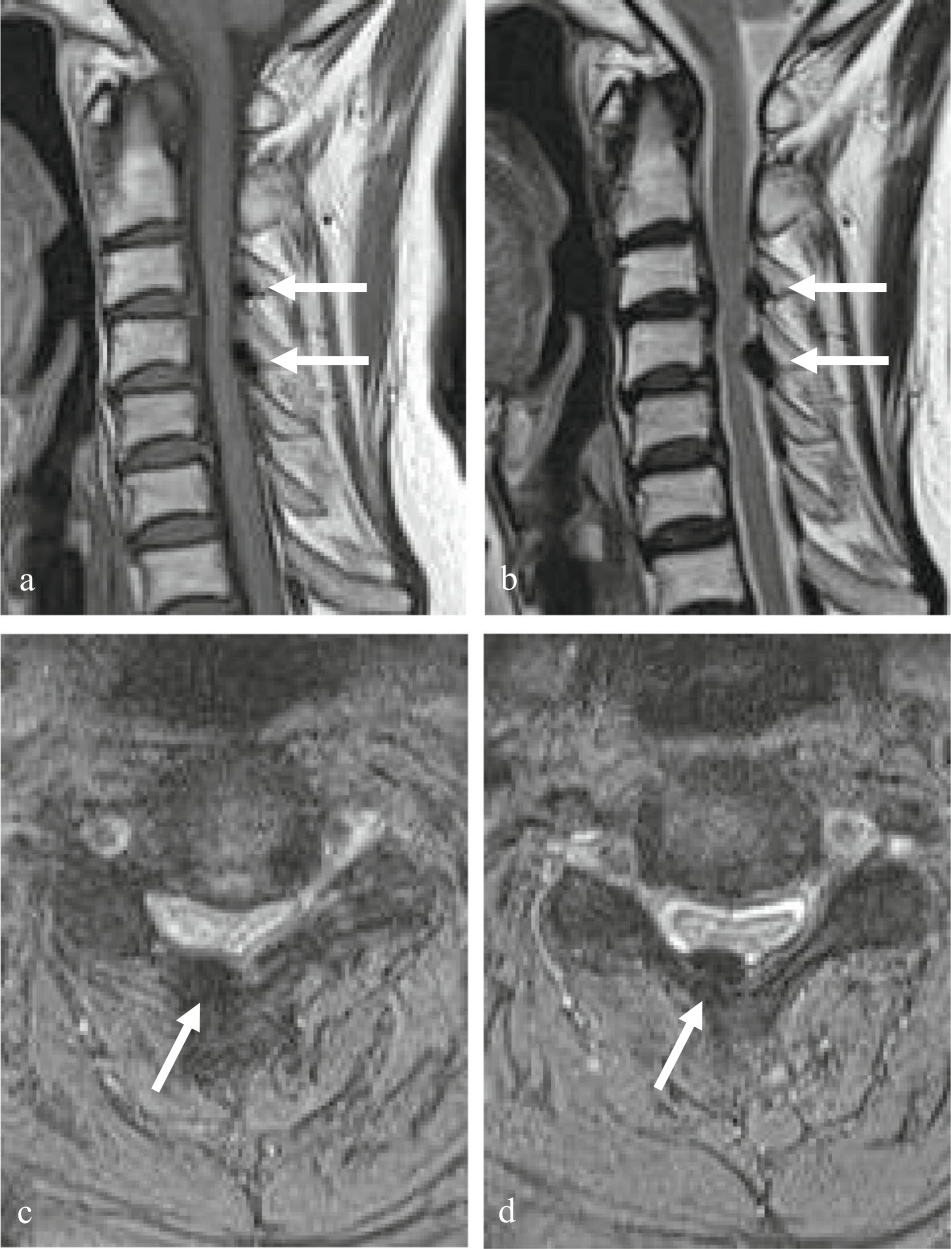
Sagittal (**a** and **b**) and axial (**c** and **d**) MRI showed spinal cord compression by dark round lesions at the laminae of C3 and C4 (white arrows)

**Fig. 6:**
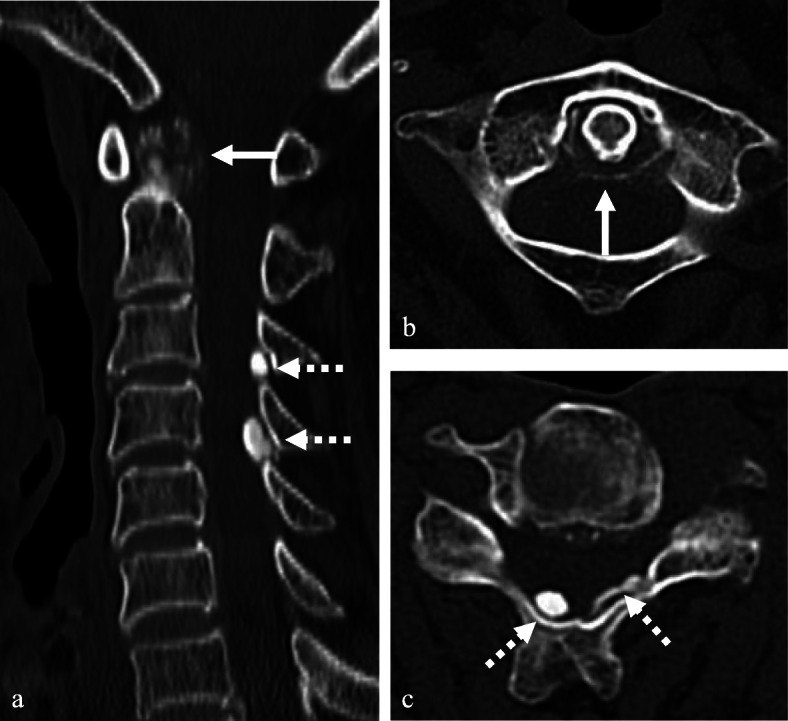
Sagittal and axial CT scans showed a calcified lesion behind the dens (**a** and **b**) (white arrows) and spinal cord compression by calcified round lesions at C3 and C4 (**a** and **c**) (dotted white arrows)

**Fig. 7:**
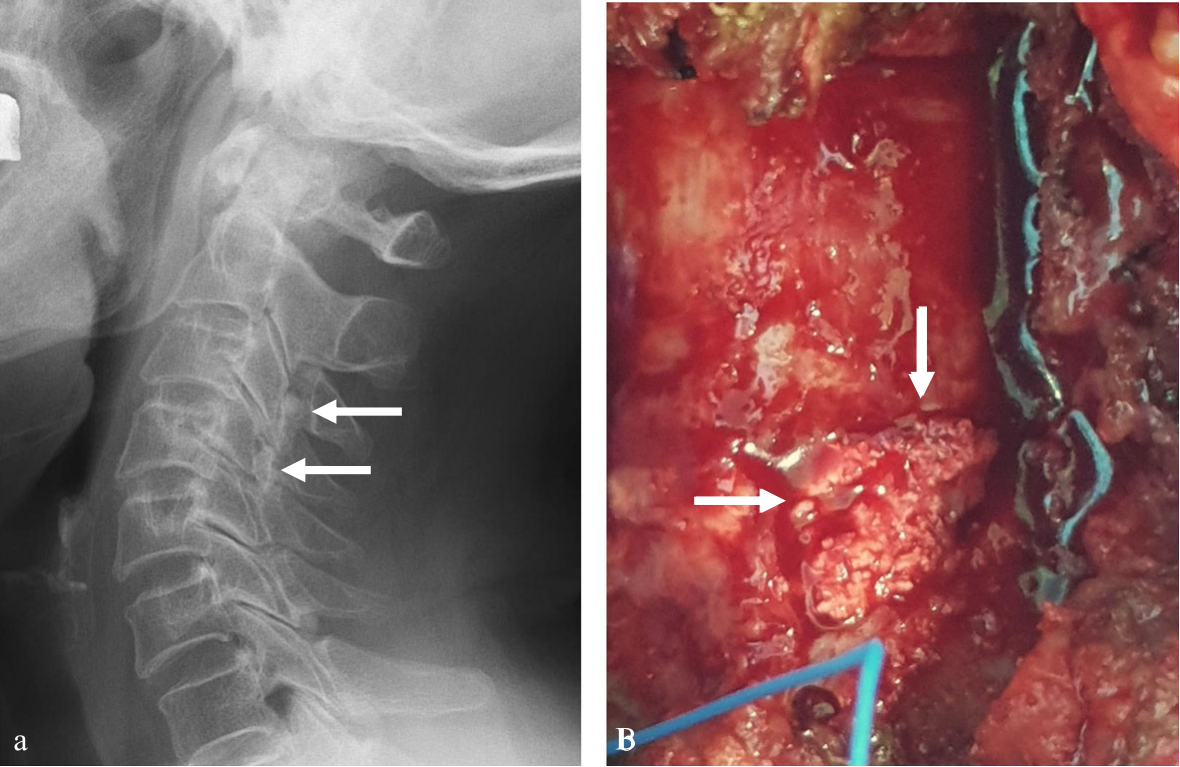
Lateral radiograph (**a**) of the cervical spine showed round calcified lesions at the laminae of C3 and C4 (white arrows). Intraoperative clinical photo (**b**) revealed CPPD deposition after total laminectomy of C3 and C4 (white arrows)

**Fig. 8:**
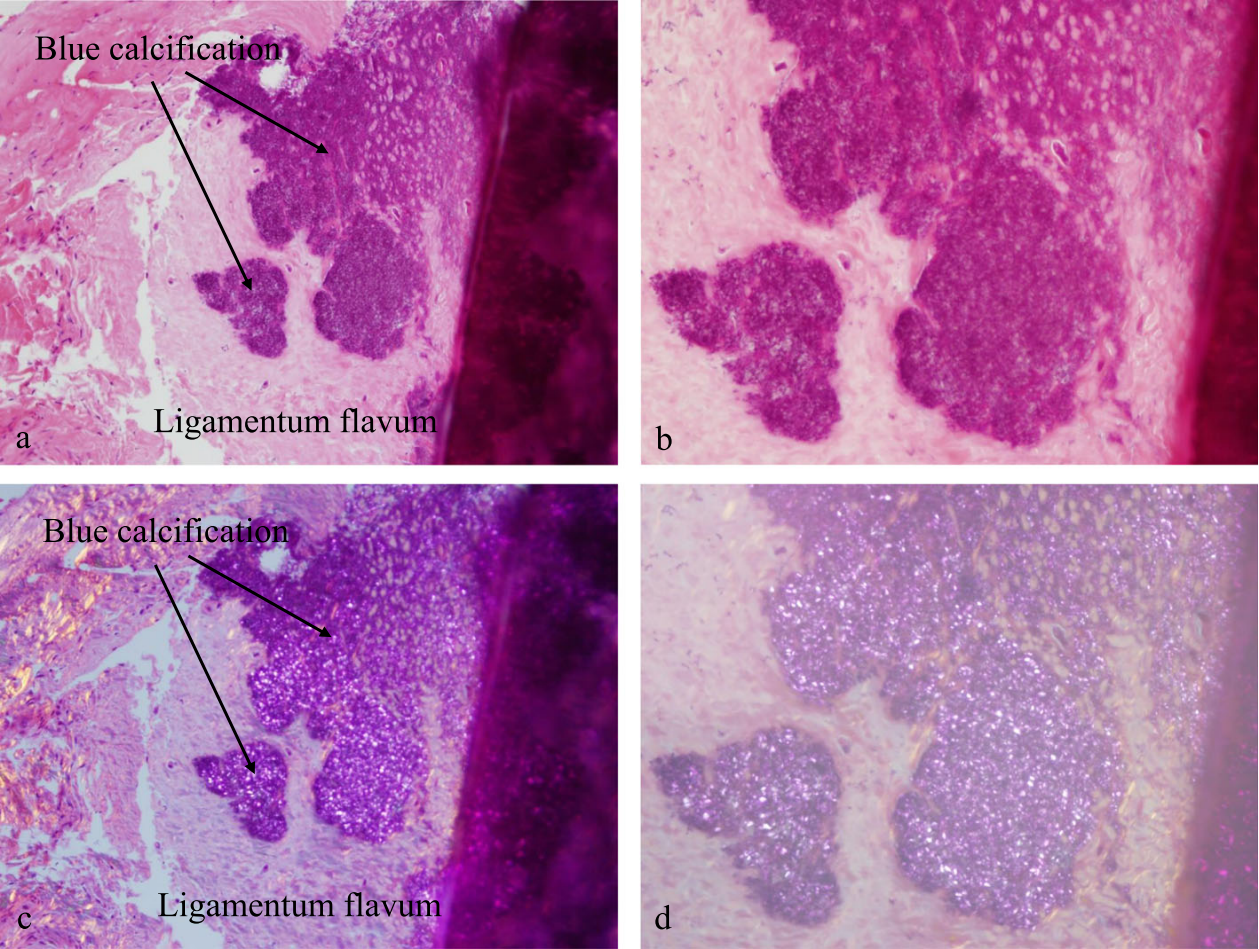
Microscopic evaluation (× 200, **a**; × 400, **b**) of the surgical specimen demonstrated degenerated ligamentum flavum with dark blue calcifications representing chondrocalcinosis. Microscopic evaluation (× 200, **c**; × 400, **d**) of the surgical specimen under polarized light showed rhomboid crystals that were positively birefringent in the blue calcification area

A 77-year-old female presented with neck pain (neck VAS score: 4), bilateral radiating arm pain (arm VAS score: 6/6), and gait disturbance for 10 days. She had no history of recent head or neck injuries. Neurological examination revealed a spastic gait, hand clumsiness, and exaggerated deep tendon reflexes in the bilateral upper and lower extremities. Pathologic Babinski sign and ankle clonus were present. Muscle strength of both lower extremities was diffusely decreased. Grip and release test was 14 times per 20 s. mJOA score was 8. However, she had no dysuria or constipation. Sagittal (Fig. 5a and b) and axial (Fig. 5c and d) MRI showed spinal cord compression by dark round lesions at the laminae of C3 and C4 which appeared as hypointense masses on both T1 and T2 weighted images. Sagittal and axial CT scans showed a calcified lesion behind the dens (Fig. 6a and b) and spinal cord compression by round calcified lesions at C3 and C4 (Fig. 6a and c). Lateral radiograph of the cervical spine showed round calcified lesions at the laminae of C3 and C4 (Fig. 7a). The patient received decompressive laminectomy of C3 and C4 and removal of round calcified lesions. An intraoperative clinical photo (Fig. 7b) showed round calcified lesions compressing the spinal cord. Microscopic evaluation (× 200, Fig. 8a; × 400, Fig. 8b) of the surgical specimen demonstrated a degenerated ligamentum flavum with dark blue calcifications representing chondrocalcinosis. Microscopic evaluation (× 200, Fig. 8c; × 400, Fig. 8d) of the surgical specimen under polarized light showed rhomboid crystals that were positively birefringent in the blue calcification area. The findings were consistent with CPPD. The patient’s myelopathic symptoms were significantly improved after surgery to mJOA of 13 with a recovery rate of 50% and showed a satisfactory surgical outcome without recurrence at a 2-year follow-up.

## Discussion and conclusions

CPPD deposition is a crystal arthropathy primarily affecting the peripheral joints, most commonly the wrist and knee. However, CPPD deposition in the cervical spine is a rare entity [1–7]. CPPD deposition in the cervical spine is an unusual cause of cervical spinal cord compression and myelopathy. Retro-odontoid CPPD deposition may lead to acute pseudogout and can be visualized as calcifications that surround the apex of the dens, and therefore, it is known as “CDS” [8–13]. This entity may manifest itself in a variety of ways, but it usually presents as acute neck pain and stiffness [8, 10, 16]. With disease progression, it may present as a mass-occupying lesion and cause progressive cervical spinal cord compression and myelopathy [11, 12].

CPPD deposition of the spine, including CDS, has been previously described, but it is very unusual in the lower cervical spine [2, 3, 5, 14, 15]. There are very few reports of cervical myelopathy caused by subaxial CPPD deposition and there has been no report on cervical myelopathy by subaxial CPPD deposition with simultaneous asymptomatic CDS at the same time. To the best of our knowledge, this is the first report on cervical myelopathy caused by subaxial CPPD deposition with simultaneous asymptomatic CDS at the same time.

The CDS is characterized by various clinical manifestations of cervical pain and neck stiffness and myelopathy. Calcification can occur at not only the cruciform ligament but also the transverse, alar and apical ligaments [8–13]. Cervical CPPD deposition may produce a unique type of neck pain from the suboccipital region to the posterior neck bilaterally with restricted motion in rotation [2, 17–21]. Furthermore, compressive cervical myelopathy can manifest, especially when CPPD deposition occurs at the ligamentum flavum [14, 15]. In each of the present cases, the patient had asymptomatic CDS. A calcified lesion behind the dens did not compress the spinal cord, and the patient complained of only mild neck pain with the neck VAS score of 3–4 without myelopathy. The patients also showed C3 and C4 CPPD deposition, resulting in spinal cord compression causing cervical myelopathy symptoms, such as hand clumsiness, gait disturbance, increased deep tendon reflexes, and pathologic reflexes. Therefore, we believe that CDS was not considered as cause of myelopathy in our two cases. Clinical symptoms are not always correlated with the radiological findings and therefore it is very important to thoroughly evaluate the difference between clinical symptoms and radiologic findings.

CT scans, the most diagnostic examination, demonstrate oval-shaped calcified lesions anterior to the laminae with clear margins [22, 23]. The lesions are observed centrally, not laterally, beneath the laminae. Similarly, in our cases, the lesion appeared to be located in the middle-to-posterior portion of the spinal canal at the C3 and C4 level, and it compressed the spinal cord posteriorly from the bases of C3 and C4 laminae [1]. On MRI, CPPD deposition manifests as a predominately hypointense area on T1 and T2 weighted images, as in the present cases [3, 24, 25]. Moreover, MRI is also useful to assess spinal cord compression or myelopathy [3, 24, 25]. Histology can confirm marked degeneration of elastic fibers about the calcium deposits showing irregular arrangement of the elastic fibers, abnormally small diameter of fragmented elastic fibers, and thick bundles of collagen fibers. Additionally, a number of chondrocytes can be found around the calcium deposition-containing matrix together with proliferation of small blood vessels and palisading histiocytes and foreign body-type giant cells in the periphery of the calcified lesion [1, 25]. In our study, microscopic evaluation demonstrated degenerative ligamentum flavum with dark blue calcifications, which represented chondrocalcinosis as rhomboid crystals that were positively birefringent in the blue calcification area under polarized light microscope, and these findings were consistent with those of CPPD.

The treatment of CPPD deposition in the cervical spine is based on clinical symptoms [1]. Conservative treatment including medication can be attempted before surgical intervention. However, surgery must be conducted for cervical myelopathy before patients suffer irreversible spinal cord damage [1, 6, 8, 11, 12]. In each of our cases, we considered conservative treatment as sufficient for the asymptomatic CDS, but surgical treatment was necessary for cervical cord compression due to subaxial CPPD deposition. Consequently, surgical removal of subaxial CPPD deposition achieved a satisfactory surgical outcome and showed a good prognosis without chance of recurrence. In general, the CPPD deposition can be removed by performing decompressive laminectomy for the segments with CPPD, as demonstrated in the second case. However, if there is an associated kyphosis and/or severe spondylosis preoperatively as in the first case, or if there is a need for an excessive decompression including facet joint, it is better to perform a combined fusion procedure for the prevention of the deterioration of symptoms caused by postoperative instability and kyphosis. However, the limitation of this study is case reports and future trials with large sample size are needed to establish our results.

In conclusion, this is the first study to report cervical myelopathy caused by subaxial CPPD deposition with simultaneous asymptomatic CDS. Surgical removal of the subaxial CPPD deposition alone achieved a satisfactory surgical outcome without recurrence.

## Data Availability

Not applicable as this is a case report.

## Abbreviations

CPPD: Calcium pyrophosphate dehydrate
CDS: Crowned dens syndrome
VAS: Visual analogue scale
mJOA: Modified Japanese Orthopaedic Association
MRI: Magnetic resonance imaging
CT: Computed tomography
